# Static or Temporal? Semantic Scene Simplification to Aid Wayfinding in Immersive Simulations of Bionic Vision

**DOI:** 10.1145/3756884.3766003

**Published:** 2025-12-04

**Authors:** Justin M. Kasowski, Apurv Varshney, Michael Beyeler

**Affiliations:** University of California, Santa Barbara, CA, USA; University of California, Santa Barbara, CA, USA; University of California, Santa Barbara, CA, USA

**Keywords:** bionic vision, virtual reality, wayfinding, scene simplification

## Abstract

Visual neuroprostheses (*bionic eyes*) aim to restore a rudimentary form of vision by translating camera input into patterns of electrical stimulation. To improve scene understanding under extreme resolution and bandwidth constraints, prior work has explored computer vision techniques such as semantic segmentation and depth estimation. However, presenting all task-relevant information simultaneously can overwhelm users in cluttered environments. We compare two complementary approaches to semantic preprocessing in immersive virtual reality: *SemanticEdges*, which highlights all relevant objects at once, and *SemanticRaster*, which staggers object categories over time to reduce visual clutter. Using a biologically grounded simulation of bionic vision, 18 sighted participants performed a wayfinding task in a dynamic urban environment across three conditions: edge-based baseline (*Control*), *SemanticEdges*, and *SemanticRaster*. Both semantic strategies improved performance and user experience relative to the baseline, with each offering distinct trade-offs: *SemanticEdges* increased the odds of success, while *SemanticRaster* boosted the likelihood of collision-free completions. These findings underscore the value of adaptive semantic preprocessing for bionic vision and, more broadly, may inform the design of low-bandwidth visual interfaces in XR that must balance information density, task relevance, and perceptual clarity.

## 1 Introduction

**Figure 1:**
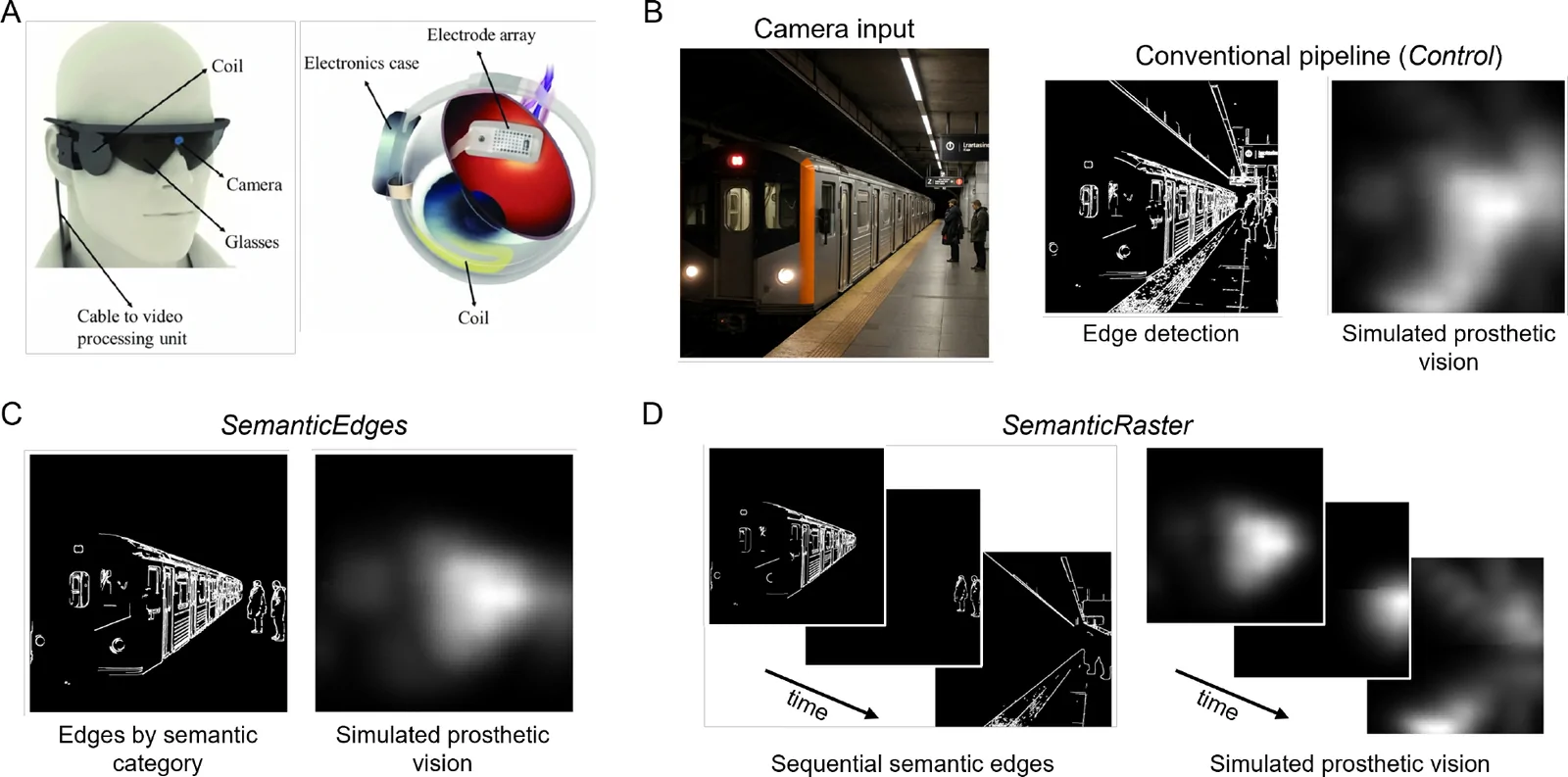
Scene simplification for bionic vision. (A) Example retinal implant (Argus II): a head-mounted camera provides visual input that is delivered as electrical stimulation through a microelectrode array in the retina (image reused under CC BY from [5]). (B) Traditional preprocessing methods, such as edge detection (*Control*), emphasize basic scene features but do not prioritize task-relevant information. (C) *SemanticEdges* enhances perception by isolating key semantic groups (e.g., pedestrians, obstacles) while suppressing irrelevant background details. (D) *SemanticRaster* extends this approach by sequencing semantic groups across frames, prioritizing hazards first to reduce clutter and improve scene understanding in dynamic environments.

By 2050, over 114 million people are expected to be living with incurable blindness, representing a major global health challenge [6]. Electronic visual prostheses, or *bionic eyes*, aim to restore rudimentary vision by electrically stimulating surviving neurons in the retina, optic nerve, or visual cortex [14, 56]. While clinically approved systems such as the Argus II (Fig. 1A) can provide functional vision for navigation and object localization [17, 33], they remain limited by low resolution, narrow fields of view, and strict safety regulations on simultaneous electrode activation.

Commercial devices typically activate only subsets of electrodes (*timing groups*) in rapid temporal succession [49], a raster-scanning approach inspired by display technology. Raster patterns are chosen heuristically and remain agnostic to scene content. Recent work suggests that checkerboard rasters, which maximize spatial distance between active electrodes, can improve clarity and task performance while complying with safety limits [28]. In parallel, preprocessing strategies such as semantic segmentation [20, 48] or depth-based cues [35, 43, 46] can highlight task-relevant information. However, even simplified images often overwhelm the user when displayed all at once under tight stimulation constraints [4].

We propose a novel content-aware raster strategy called *SemanticRaster*, which bridges these two perspectives. Rather than activating spatial strips or checkerboards, the system cycles through semantic groups over time: for example, first displaying hazards like cars or bicycles, then pedestrians, then structural elements (Fig.1D). This approach aims to reduce clutter and direct attention to task-relevant features while maintaining context across frames. The prioritization of object categories is flexible and ideally co-designed with blind users [10, 37, 44], offering a foundation for temporally adaptive encoding that reflects user needs and task demands.

Because no commercial retinal implants are currently available (and clinical testing is constrained by risk, device heterogeneity, and small sample sizes), direct evaluation of raster strategies in end users is infeasible. Simulated prosthetic vision (SPV) in immersive virtual reality (VR) provides a powerful alternative [12, 21, 27], enabling repeatable testing of design strategies in realistic settings. Here we use BionicVisionXR [27], an open-source VR platform with gaze-contingent rendering and psychophysically grounded models of phosphene appearance [2], temporal dynamics [25], and spatial summation [24], to emulate how a future implant may respond to head and eye movements.

In this study, 18 sighted participants completed a wayfinding task through a cluttered virtual town square using SPV. While sighted participants cannot model long-term perceptual learning, they enable controlled, within-subject comparisons that are impractical in implant users [3]. Gaze-contingent rendering allowed us to emulate the visual experience of a head-mounted camera system interacting with retinal stimulation. Code is available at https://github.com/bionicvisionlab/2025-VRST-SmartRaster.

Our work makes three key contributions:

- We introduce *SemanticRaster*, a content-aware raster strategy that sequences semantic groups over time, offering a new method for reducing clutter while preserving context under tight stimulation constraints.
- We conduct a controlled user study in immersive VR that systematically compares static and temporally adaptive semantic encoding strategies using realistic phosphene simulations and dynamic obstacles.
- We show that static and temporally sequenced semantic simplification confer complementary benefits (higher completion and lower collision rates, respectively), providing design guidance for bandwidth-limited XR and next-gen bionic-vision interfaces.

## 2 Background

Several classes of bionic vision systems are under development, including retinal, optic nerve, and cortical implants. Retinal devices such as the Argus II [33], Alpha-IMS [51], and suprachoroidal systems [54] represent the most clinically advanced, while next-generation systems such as PRIMA [38], ICVP [26], and Neuralink’s cortical array [36] aim to improve resolution and usability through denser electrode layouts and flexible implantation strategies.

Most of these systems rely on an external visual processing unit (VPU) to convert real-time video into stimulation patterns for the implanted electrode array. While electrical activation can elicit *phosphenes* (discrete points of light), the resulting vision remains highly degraded: resolution is limited [15, 57], visual fields are narrow (e.g., 10×20° in Argus II) [33], and percepts are variable and often distorted by biological factors [2, 50]. Safety limits on simultaneous electrode activation further constrain effective resolution, even as newer devices push electrode counts into the hundreds.

As a result, users often describe bionic vision as unreliable, effortful, and situationally useful at best [37]. Navigation and scene understanding remain especially challenging, as the limited field of view (FoV) necessitates continuous head scanning to piece together a coherent sense of the environment [13]. Most current systems ignore eye movements [8], further complicating perceptual stability.

To improve usability and support greater independence, future prosthetic systems must not only improve hardware, but also intelligently preprocess visual input. This includes prioritizing task-relevant information, reducing clutter, and adapting to the user’s context and behavior [4]. SPV in immersive VR has emerged as a powerful tool to prototype and evaluate such strategies, enabling rapid iteration without the need for implantable hardware.

## 3 Related Work

SPV in immersive VR has emerged as a powerful testbed for evaluating encoding strategies prior to clinical deployment. Sighted participants can act as “virtual patients,” experiencing key constraints of bionic vision (i.e., reduced resolution, limited FoV, phosphene blur, and temporal distortions) without the variability introduced by long-term adaptation or device-specific idiosyncrasies [27, 43, 53]. While not a substitute for real-world testing, SPV enables controlled, repeatable, within-subject comparisons that are impractical in clinical studies, especially during early-stage prototyping.

Early work on preprocessing emphasized edge detection and contrast enhancement to make scene structure more perceptible [12, 55], though these methods lacked adaptability to tasks or environments. For example, Dagnelie et al. [12] found that edge enhancement improved detection of large objects but broke down in cluttered scenes, while Vergnieux et al. [55] showed that contrast filtering highlighted scene boundaries at the cost of fine detail needed for navigation.

More recent studies have applied computer vision to highlight task-relevant features. Semantic labeling can aid object recognition in SPV but displaying all classes simultaneously increases clutter [20]. Depth-based preprocessing emphasizes nearby hazards and improves obstacle avoidance [35, 46], but sometimes suppresses distal cues. Thorn et al. [53] showed that obstacle avoidance performance degraded sharply as clutter increased, even with edge enhancement, and Rasla et al. [43] found that relative-depth encoding could improve mobility only under tightly controlled conditions. Together these findings highlight the trade-off between clarity and informational value.

To address this, time-multiplexed rendering strategies have been explored, though only sparingly. Kasowski et al. [28] compared rastering strategies in SPV and found that a checkerboard pattern yielded higher accuracy for letter recognition and motion discrimination than row-wise, column-wise, or random rasters. However, their stimuli were simple and static, leaving open whether similar benefits extend to cluttered navigation tasks. Early SPV studies relied on oversimplified visual models [12], but more recent work has introduced psychophysically validated phosphene simulations that incorporate fading, spatial distortion, and gaze contingency [2, 27]. Still, these advances have largely lacked temporally adaptive encoding aligned with users’ moment-to-moment navigation goals.

Our work builds on this foundation by integrating (i) a biologically grounded, gaze-contingent phosphene simulation; (ii) semantically informed image processing; and (iii) a novel raster strategy that sequences object categories based on task relevance. Unlike prior approaches that treat semantic segmentation as static, *SemanticRaster* encodes temporal prioritization to emphasize critical cues (e.g., moving obstacles) while minimizing clutter.

Though motivated by bionic vision, our framework may offer general-purpose strategies for temporally adaptive scene simplification in constrained visual displays. By combining perceptual realism, gaze contingency, and task-aware encoding, this work advances the design of real-time, user-centered interfaces for immersive and assistive technologies alike.

## 4 Methods

### 4.1 Participants

Eighteen participants with normal or corrected-to-normal vision (11 female, 7 male; ages 18–40; *M* = 25.04, *SD* = 5.72) were recruited for this study. Participants were undergraduate students recruited from the research participant pool at the University of California, Santa Barbara, and served as “virtual patients” [27] in SPV experiments.

Prior experience with VR varied: five participants had never used VR, while the remaining 13 reported familiarity with the technology, ranging from 1 to over 20 prior sessions. To minimize risks of discomfort, participants with known sensitivity to flashing lights or motion sickness were excluded during the screening process.

The study adhered to the principles of the Declaration of Helsinki and was approved by the Institutional Review Board at the University of California, Santa Barbara.

### 4.2 Simulated Prosthetic Vision

We utilized the open-source Unity toolbox BionicVisionXR (https://github.com/bionicvisionlab/BionicVisionXR), to simulate prosthetic vision within an immersive VR environment. Participants viewed stimuli through an HTC VIVE Pro Eye head-mounted display, with phosphene appearance modeled using psychophysically validated simulations [2, 18, 23]. These simulations incorporated spatiotemporal dynamics, including phosphene elongation and fading due to axonal pathways [24] (Section 4.2.1), as well as persistence and decay effects based on charge accumulation dynamics [23] (Section 4.2.2).

To approximate the visual experiences of retinal prosthesis users, the VR environment featured gaze-contingent rendering (Section 4.2.3), dynamically updating scene content based on participants’ head and eye movements. This ensured a realistic and interactive simulation of prosthetic vision.

We simulated a 10 × 10 epiretinal electrode array centered over the fovea, inspired by the Argus II implant [33]. Electrodes were modeled as point sources with 400 μm spacing, consistent with current-generation retinal prostheses. All simulations were rendered on a high-performance desktop computer (Intel i9–11900k, 64GB RAM, Nvidia RTX3090) and wirelessly transmitted to the head-mounted display.

**Figure 2:**
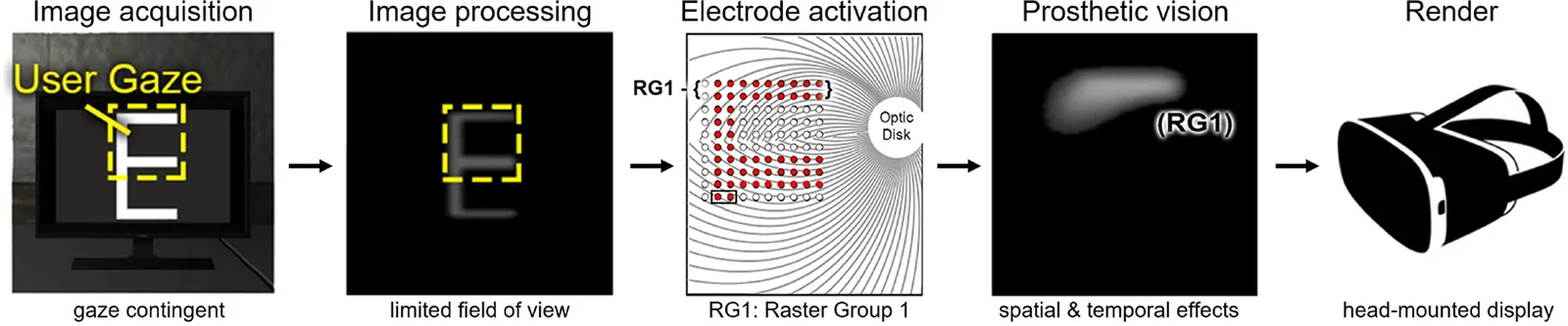
Simplified overview of the SPV pipeline. Unity’s virtual camera captured scenes while tracking gaze position (“Image acquisition”). Frames underwent scene simplification, scaling, and grayscale conversion to mimic preprocessing by a visual processing unit (“Image processing”). Electrode activation levels were derived from pixel intensities, with temporal sequencing strategies grouping electrode activations over time (“Electrode activation”). The example illustrates grouped activation of the top two rows of electrodes. Spatial distortions were modeled using an axon map, and temporal effects like fading and persistence were integrated to simulate prosthetic vision (“Prosthetic vision”). The resulting percept was rendered to participants via a head-mounted display (“Render”).

This setup balances generalizability with alignment to near-future prosthetic technologies, providing a robust platform for evaluating visual preprocessing strategies in SPV. The entire SPV workflow was thus as follows (Fig. 2):

- *Image acquisition:* Unity’s virtual camera captured a 60° FoV, rendered at 90 Hz.
- *Image processing:* Frames were downscaled to 200 × 200 pixels, converted to grayscale, and smoothed with a 3 × 3 Gaussian kernel.
- *Electrode activation:* Pixel intensities nearest to each electrode were used to compute activation levels.
- *Spatiotemporal effects:* Phosphene shapes were modeled using the axon map model [2, 18], simulating elongated phosphenes aligned with retinal ganglion cell axons. A temporal model [23] simulated phosphene fading and persistence by accounting for charge accumulation and decay.
- *Gaze-contingent rendering:* The implant location dynamically shifted based on gaze position, ensuring the scene remained aligned with participants’ fixation.

#### 4.2.1 Spatial Distortions.

The shape of phosphenes in epiretinal devices is influenced by the retinal ganglion cell axons, which traverse the retina in curved paths [2, 45]. We used the axon map model to simulate these distortions [2, 18]. Each electrode activated a region of the retina defined by Gaussian falloff parameters ρ (spread) and λ (elongation). The instantaneous brightness $b_{I}$ of each pixel (r,θ) in the percept was computed according to:

(1) $$b_{I}=\underset{p\in R(\theta )}{max}\sum_{e\in E}exp\left(\frac{-d_{e}^{2}}{2\rho^{2}}+\frac{-d_{\text{soma}}^{2}}{2\lambda^{2}}\right),$$

where R(θ) is the path of the axon terminating at retinal location (r,θ), p is a point along the path, $d_{e}$ is the distance from p to the stimulating electrode e, and $d_{\text{soma}}$ is the distance along the axon from p to the cell body. Spatial distortions were modeled using medium levels of elongation and spread, as reported in earlier psychophysical studies [2], with ρ=200μm (spread) and λ=400μm (elongation). These parameters were selected to represent typical distortions experienced by prosthesis users, balancing realism and perceptual clarity for the purposes of the study. By keeping these parameters constant across conditions, we ensured that observed differences in performance were attributable to the preprocessing strategies rather than variations in spatial distortions.

#### 4.2.2 Temporal Distortions.

To model temporal dynamics, we used a simplified variant of the Horsager et al. [23] model, which incorporates two coupled leaky integrators to simulate neural desensitization n(t) and phosphene brightness b(t). The governing equations were:

(2) $$\frac{dn(t)}{dt}=-\tau_{n}n(t)+b_{I}(t),$$

(3) $$\frac{db(t)}{dt}=-\tau_{b}b(t)-\alpha n(t)+b_{I}(t),$$

where $b_{I}(t)$ was the instantaneous brightness (from the spatial model) calculated at time t. Parameter values ($\tau_{n}=0.2\;s$, $\tau_{b}=5\;s$, and α=0.2) were fitted to reproduce temporal fading and persistence effects reported by Subject 5 of Pérez Fornos et al. [41] (see their Figure 4).

#### 4.2.3 Gaze-Contingent Phosphene Rendering.

Modern retinal prostheses rely on head-mounted cameras, so visual input is head-centered rather than eye-centered. To simulate this realistically, we implemented gaze-contingent rendering that re-centered the implant display on the participant’s fixation point in real time. Using the HTC Vive Pro Eye, each video frame was shifted according to gaze position, ensuring phosphenes were rendered in retinal coordinates and moved naturally with the eyes. This step is critical for reproducing perceptual effects such as fading, streaking, and local adaptation [39, 41]; without it, stimulation would remain fixed to the screen, producing smeared or distorted percepts during eye movements.

Our setup achieved a mean eye-tracking precision of 1.9°, with 94 % of samples within 5° of the target (see Supplementary Material). Gaze-contingent stimulation is increasingly recognized as essential for biologically plausible SPV [8, 28, 39], and future implants are expected to support it through onboard sensors or external eye trackers.

### 4.3 Scene Simplification Strategies

**Figure 3:**
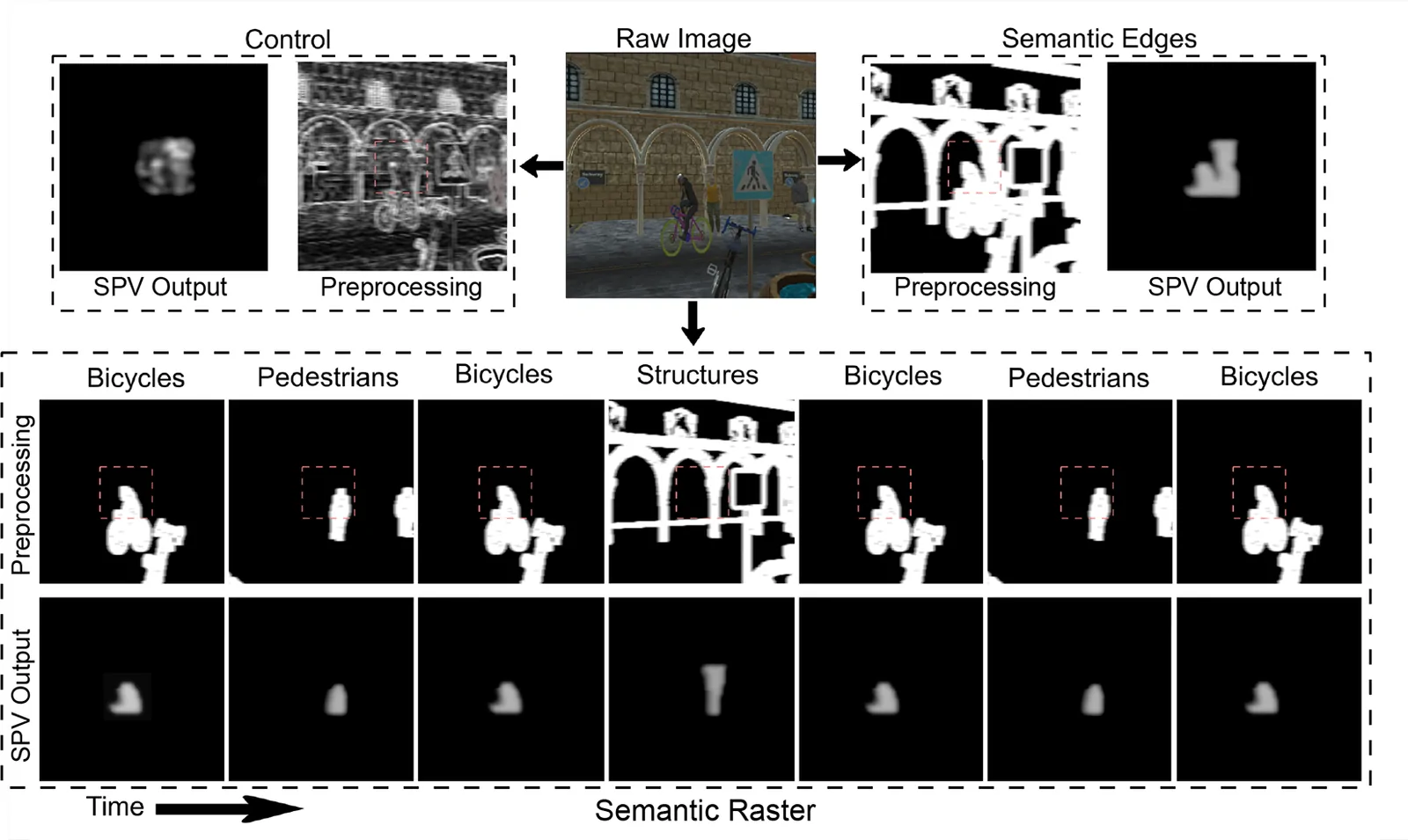
Scene simplification strategies tested in the study. The raw RGB image (top center) was processed using three methods. *Control* (top left) applied a 3 × 3 Sobel filter to highlight edges without prioritizing task-relevant features. *SemanticEdges* (top right) used semantic segmentation and a 7 × 7 kernel to enhance edges of selected classes (bicycles, pedestrians, and structures). *SemanticRaster* (bottom) grouped semantic categories and displayed them sequentially over time, cycling semantic groups over time with higher update rates for dynamic objects. The final panel(s) for each method show(s) simulated prosthetic vision (SPV) output rendered in retinal coordinates; red dashed boxes mark the limited field of view of the SPV rendering.

To evaluate the effect of different scene simplification strategies on wayfinding performance, the SPV system rendered visual input using three distinct strategies:

- *Control:* This baseline condition applied a standard 3 × 3 Sobel kernel to the input for edge detection. While effective for emphasizing structural boundaries, this method lacked task-specific prioritization, often resulting in a cluttered visual field that could overwhelm users in complex environments.
- *SemanticEdges:* A semantically informed edge filter that emphasized high-priority objects (e.g., pedestrians, bicycles, and structural features) based on scene understanding. A 7 × 7 Sobel kernel enhanced edges while suppressing irrelevant background details, reducing clutter and emphasizing salient features.
- *SemanticRaster:* A novel strategy that combined semantic segmentation with temporal prioritization. Rather than displaying all object classes simultaneously, this mode cycled through key object categories over time (200 ms per class), repeatedly displaying bicycles, then pedestrians, then structural edges. This schedule aimed to reduce crowding and improve perception under the low-resolution constraints of SPV (Fig. 3).

#### 4.3.1 Task Relevance and Raster Schedule.

An object class was deemed *task-relevant* if (i) the task involved interacting with, avoiding, or locating it, and (ii) failure to perceive it impaired performance (e.g., more collisions or timeouts). Classes were identified with input from a blind consultant and an O&M specialist. For wayfinding, this yielded three key classes (bicycles, pedestrians, and structural edges) in that order of importance. *SemanticRaster* reflected this priority by allocating equal temporal slots (200 ms) to each class, with higher-ranked categories recurring more frequently.

This framework generalizes: a street-crossing task might prioritize cars and crosswalks, while an indoor task might emphasize doors and furniture. The rastering mechanism is unchanged; only the class set and ordering vary, based on structured input from end users and task pilots [16, 22].

#### 4.3.2 Stimulation Constraints.

Although these strategies prioritized relevant information, they still required stimulating many electrodes per frame, risking safety limits. To mitigate this, all strategies used a checkerboard raster pattern shown to be perceptually effective [28], alternating activation across the grid to avoid simultaneous neighbors. Cycling at 90 Hz to match the headset refresh, this approach exploited temporal integration to yield coherent percepts while minimizing crosstalk and phosphene fusion.

### 4.4 Task & Environment

**Figure 4:**
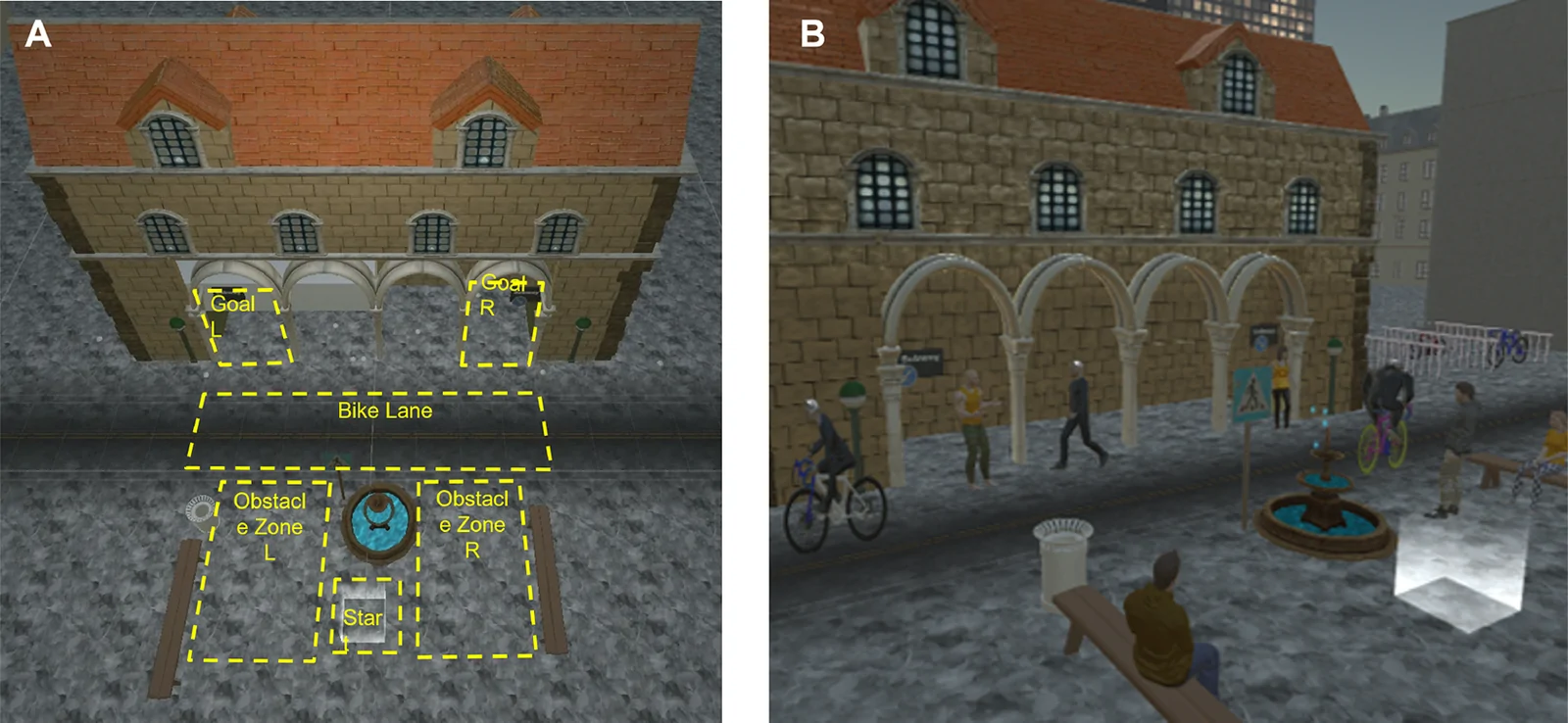
Task environment in the simulated urban square. (A) Overhead view showing the two possible goal locations (“Goal L” and “Goal R”), the designated bike lane for spawning cyclists, and two obstacle zones (“Obstacle Zone L” and “Obstacle Zone R”) used for pseudorandom placement of static objects on each trial. (B) Example first-person view of one trial instantiation, with spawned pedestrians, cyclists, and static obstacles (e.g., benches, fountain). The white square marks the starting position. Participants were instructed to navigate to the assigned goal entrance while avoiding collisions. Trials ended successfully when the participant reached the correct entrance, or terminated early due to a bicycle collision or exceeding the 50 s time limit.

Participants completed an ambulatory wayfinding task in a SPV environment modeled after a 7.5 m × 7.5 m urban square (Fig. 4). The square featured dynamic obstacles (bicycles, pedestrians) and static ones (benches, lampposts), with spatialized sound for realism. The objective was to walk from the starting position (in front of the fountain) to one of two subway entrances while avoiding collisions. Participants navigated by walking in the tracked space.

Static obstacle configurations (e.g., benches, standing pedestrians) were drawn from a set of predefined layouts, with one pseudo-randomly selected per trial to increase variability. This ensured that all trials were comparable in difficulty while preventing participants from memorizing obstacle layouts. Dynamic obstacles followed predefined paths, but their speed and timing were randomized across trials to prevent memorization. The SPV simulation reflected key constraints of current bionic vision systems, including a reduced FoV (14.6° × 14.6°) and a phosphene resolution of 10 × 10 electrodes, rendered in a gaze-contingent and temporally dynamic manner.

To ensure participant safety during the ambulatory task, the virtual environment was overlaid onto a large, obstacle-free physical space. A trained experimenter continuously monitored participants and was ready to intervene if needed. The VR headset (HTC Vive Pro Eye) was connected to the rendering computer via a high-bandwidth wireless adapter, eliminating tethering cables that could pose tripping hazards during walking. This wireless link introduced no perceptible latency (all rendering remained at 90 Hz), and thus did not affect task performance.

#### 4.4.1 Training Phase.

Participants completed a structured training session to acclimate to the SPV environment and task mechanics. The session included five rounds in a simplified virtual scene with both static and dynamic obstacles. The first four rounds used normal vision. The final round introduced SPV, including temporal distortions and one of the three scene simplification modes (Control, SemanticEdges, or SemanticRaster), corresponding to the participant’s upcoming block. Participants practiced navigating and intentionally colliding with virtual objects (e.g., trashcans, bicycles) to experience the auditory and visual collision feedback. To avoid double-counting, a cooldown period suppressed additional collision registration until the participant had moved at least 0.25 m away (see Supplementary Material).

#### 4.4.2 Experimental Procedure.

The experiment followed a within-subjects block design in which participants completed all three strategies (*Control*, *SemanticEdges*, and *SemanticRaster*). The *Control* block was always presented first to prevent carryover: exposure to semantic overlays can induce head-scanning strategies that would inflate baseline performance. The two semantic blocks were counterbalanced. Each block consisted of 10 trials, for a total of 30 trials per participant.

In each trial, participants had up to 50 s to walk to the assigned subway entrance while avoiding collisions. A trial ended successfully when the goal was reached, or failed if the participant collided with a bicycle or exceeded the time limit. A countdown timer appeared with 10 seconds remaining to discourage idling and maintain consistent time pressure.

### 4.5 Data Collection & Analysis

Performance was assessed on five metrics: (i) *Task success*: reaching the assigned entrance within 50 s (bicycle collisions ended the trial; other collisions did not); (ii) *Collision-free completion*: success with zero collisions; (iii) *Collision rate*: total collisions per trial, split by obstacle type; (iv) *Completion time*: time to goal for successful trials; (v) *Task difficulty*: block-wise self-ratings on a 10-point Likert scale.

Data were analyzed with mixed-effects models matched to outcome type. Unlike mixed ANOVA, GLMMs provide the appropriate link functions for binary/count data and accommodate participant-level random effects. All models included a fixed effect of Condition (*Control*, *SemanticEdges*, *SemanticRaster*) and a random intercept for SubjectID. Because *Control* always came first, we tested for learning by adding a centered trial index and Condition × TrialIndex interaction; these did not improve model fit. For difficulty, block order was included as a covariate; main effects were unchanged.

Binary outcomes (e.g., success) were fit with logistic GLMMs, Success ~ Condition+TrialIndex+(1+TrialIndex | SubjectID). Collision counts used Poisson GLMMs, Collisions ~ Condition+TrialIndex + (1 | SubjectID); overdispersion was checked with DHARMa and, where present, results were confirmed with Negative Binomial GLMMs (see Supplementary Material). Completion times were modeled with linear mixed-effects regression, Time ~ Condition + TrialIndex + (1 + TrialIndex | SubjectID). Difficulty ratings (ordinal 1–10) used cumulative link mixed models, Difficulty ~ Condition + Order + (1 | SubjectID).

Models were fit in R (lme4 for LMM/GLMMs, glmmTMB for NB fits, and ordinal::clmm for cumulative link models); post-hoc contrasts were obtained with emmeans, Tukey-corrected. A simulation-based sensitivity analysis (Supplementary Material) indicated that the within-subject design (18 participants × 10 trials/condition) had 80% power to detect medium-to-large effects on task success, but lower power (30–57%) for smaller changes in collision rates.

## 5 Results

### 5.1 Task Success

**Figure 5:**
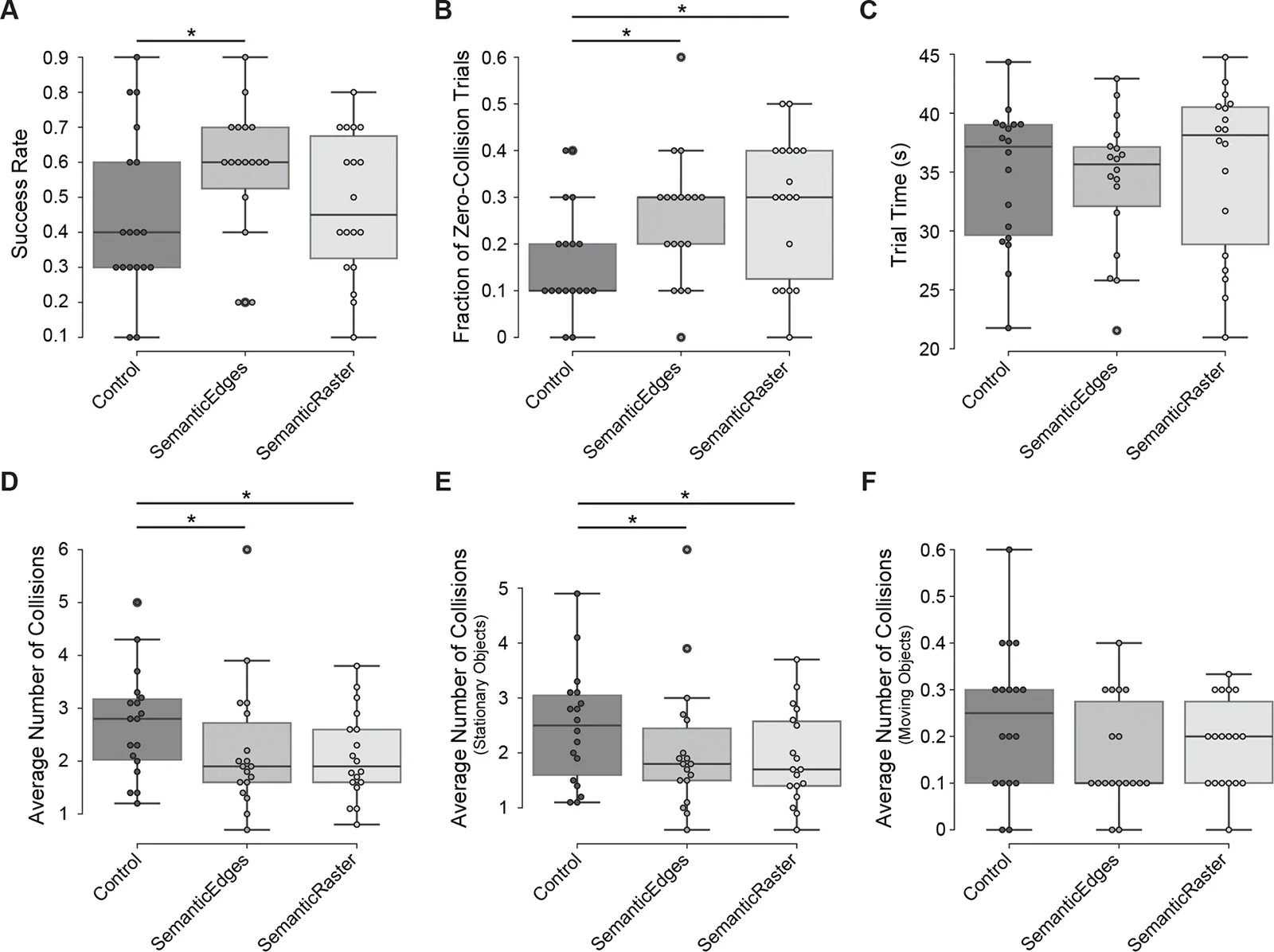
Task performance metrics across conditions. (A) Success rate: proportion of trials completed without collisions or time-outs. (B) Fraction of successful zero-collision trials. (C) Trial completion time in seconds. (D) Average number of collisions per trial. (F) Average number of collisions involving static structures (e.g., fountain, bench, standing pedestrians). (E) Average number of collisions involving moving obstacles (i.e., bicycles). Each point represents a participant; boxplots show median, interquartile range, and range. Statistical significance between conditions is denoted by * (*p* < .05).

We first examined the impact of scene simplification on task success, defined as reaching the goal before the timer expired (Fig. 5A). A generalized linear mixed-effects model (GLMM) with fixed effects of Condition and centered TrialIndex, and by-subject random intercepts and learning slopes, revealed a significant benefit for the *SemanticEdges* condition: participants were 1.84 times more likely to complete the task successfully compared to the *Control* baseline (*β* = 0.61 ± 0.24, *z* = 2.59, *p* = .009). *SemanticRaster* showed a smaller, non-significant improvement (*β* = 0.27, *p* = .24). There was a modest learning effect across trials (*β* = 0.045, *z* = 3.99, *p* < .001), but no significant interaction between condition and trial index ($\chi^{2}(2)=0.17$, *p* = .92), suggesting that the scene simplification effects were stable over time.

To evaluate whether simplification also led to cleaner navigation, we examined the odds of completing a trial collision-free (Fig. 5B). A separate GLMM (logit link) indicated that both *SemanticEdges* and *SemanticRaster* increased the likelihood of a clean run compared to the baseline (*SemanticEdges*: OR = 1.8, *p* = .086; *SemanticRaster*: OR = 2.1, *p* = .018), independent of trial index (*p* > .9), suggesting that even when overall success rates are similar, *SemanticRaster* may help users navigate more cleanly when successful.

Completion time did not vary significantly by condition or trial index (all |*t*| < 1.3, *p* > .25), indicating that these gains in accuracy were not simply due to participants slowing down (Fig. 5C). Timeouts were rare, occurring on only 10 out of 540 trials (< 2%), further indicating that participants generally completed the task within the allotted time regardless of condition.

A Condition × TrialIndex interaction term was added to each model to test whether learning differed between strategies. Across all three outcomes (success, collision counts, trial time), the interaction was non-significant (all *p* > .50), indicating that participants improved (or plateaued) at comparable rates under *SemanticRaster* and *SemanticEdges*. Thus, *SemanticRaster*’s cleaner-run advantage does not appear to hinge on an extended learning period.

### 5.2 Collision Rates

To better understand error patterns, we analyzed collision counts using Poisson GLMMs (Fig. 5D). Both *SemanticEdges* and *SemanticRaster* significantly reduced total collisions compared to *Control*, by 21% and 26%, respectively (*SemanticEdges*: *β* = −0.236, *p* = .009; *SemanticRaster*: *β* = −0.303, *p* = .001). No significant difference emerged between the two smart strategies (*β* = 0.067, *p* = .77), and collision rates remained stable across trials (*p* = .49).

Breaking down collisions by object type revealed that these improvements were driven by reductions in contact with static obstacles (Fig. 5E). Both *SemanticEdges* and *SemanticRaster* significantly decreased stationary collisions relative to *Control* (*SemanticEdges*: −18%, *β* = −0.203, *p* = .035; *SemanticRaster*: −26%, *β* = −0.302, *p* = .003). Again, the two smart modes did not differ significantly (*p* = .61), and there was no effect of trial index (*p* = .12).

Collisions with moving obstacles (e.g., cyclists) were rarer overall (Fig. 5F), but a marginal trend suggested that *SemanticEdges* may reduce such collisions relative to baseline (*β* = −0.443, *p* = .067); the effect for *SemanticRaster* was smaller and non-significant (*β* = −0.324, *p* = .17). Trial index showed a weak trend toward improvement (*p* = .055), but participant-level variance was negligible (singular fit). These results suggest that smart simplification is more effective for managing static than dynamic hazards.

Taken together, these findings indicate that improved performance was primarily driven by the simplification strategies themselves, rather than by learning across trials.

### 5.3 Perceived Difficulty

**Figure 6:**
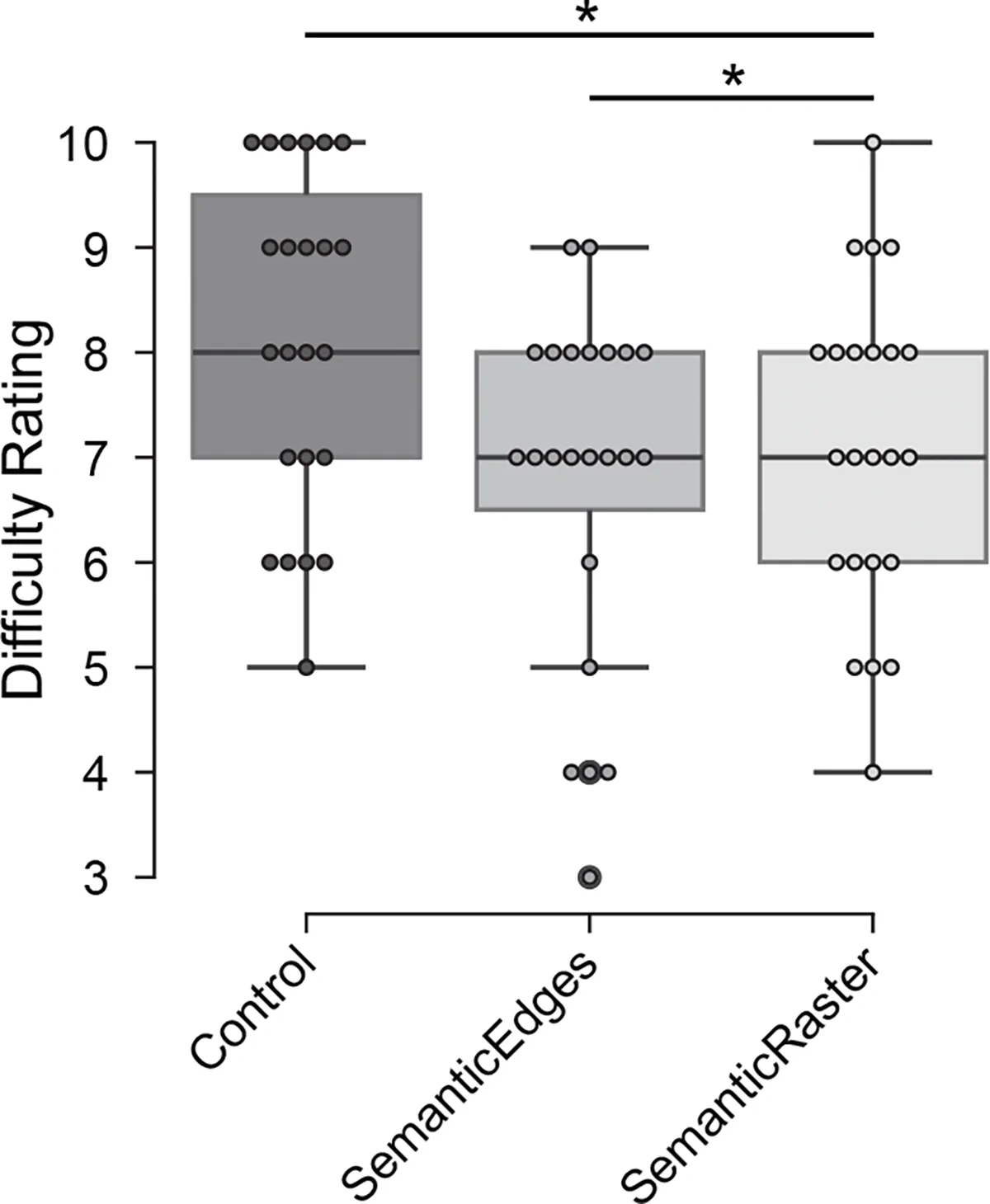
Difficulty ratings: Participants rated each condition’s difficulty on a 10-point Likert scale (1 = very easy, 10 = very hard). Both smart strategies significantly reduced perceived difficulty compared to *Control* (* *p* < .05).

Finally, after completing all trials of a given condition, participants rated its difficulty on a 1–10 scale (Fig. 6). A cumulative link mixed model revealed a significant effect of condition: both *SemanticEdges* (*β* = −1.66, *p* = .004) and *SemanticRaster* (*β* = −1.47, *p* = .010) were perceived as less difficult than *Control*. There was also a trend for later blocks to be rated as easier overall (*β* = −0.48, *p* = .085). Pairwise contrasts on the latent scale confirmed these findings: both smart modes were rated significantly easier than *Control* (*SemanticEdges*: *p* = .012; *SemanticRaster*: *p* = .027), but did not differ from each other (*p* = .94). These subjective ratings align with the objective performance improvements observed above.

## 6 Discussion

This study evaluated two semantic scene simplification strategies (*SemanticEdges* and *SemanticRaster*) for wayfinding in SPV. Both improved outcomes over a conventional baseline, but in different ways: *SemanticEdges* increased task success, whereas *SemanticRaster* improved collision-free completions. These complementary benefits underscore the value of semantically driven preprocessing.

### 6.1 Complementary Roles of Static and Temporal Simplification

A static overlay of all task-relevant classes supported global awareness, yielding more completed trials (Fig. 5A). Sequencing those classes reduced clutter, resulting in fewer collisions (Fig. 5B) and lower self-reported effort (Fig. 6). Presenting everything at once maximizes information but risks crowding, whereas staggering lightens instantaneous load but hides context briefly. Which strategy is preferable depends on what “failure” means: missing an exit (*SemanticEdges* helps) vs. clipping a hazard (*SemanticRaster* helps).

Neither strategy harmed baseline performance, and participants acclimated within a few trials. Across 30 trials we observed only modest learning, and difficulty ratings dropped by 1.5–2 points for both smart strategies. This rapid uptake is encouraging, given that perceptual learning in implant users is often measured in weeks or months [11, 13, 52].

### 6.2 Relevance for Temporally Multiplexed Implants

Modern prostheses already use raster-like stimulation because safety rules restrict simultaneous electrode activation [33]. Our *SemanticRaster* shows that this temporal budget can convey task semantics: rather than sweeping the array spatially, firmware could sweep *through* semantic layers (hazards → landmarks → context). Although our experiment used a three-layer schedule, the principle of content-aware time-division aligns with calls for adaptive stimulation policies tailored to task and context [4]. The specific categories are not universal (they were chosen for this task with input from a blind consultant and O&M specialist) and future co-design studies must refine categories, priorities, and update rates for different applications.

### 6.3 Connections to Bandwidth-Limited XR

Low-bandwidth can be defined along two axes: (i) stimulation bandwidth, limited by the fraction of electrodes active per frame and total pulse rate, and (ii) compute/throughput budget for preprocessing. In our SPV, a 10 × 10 checkerboard raster activated at most 50 electrodes per subframe at 90 Hz, i.e., ≤50% duty cycle. *SemanticRaster* respects this budget by time-dividing semantic layers rather than adding concurrent activity. On the compute side, our pipeline ran at 90 Hz on a desktop; deployable systems could use lightweight segmentation or belt-worn VPUs for real-time performance.

Similar constraints arise in AR, telepresence, and low-vision aids, where pixel budgets are limited not only by hardware (microdisplay resolution, battery, link bitrate) but also by attention [19, 40]. XR systems often reduce peripheral resolution, drop frames, or stream sparse features when bandwidth degrades. Our results suggest that multiplexing entire semantic layers is another viable strategy when clutter is the bottleneck, consistent with the view of bionic vision as a form of neuroadaptive XR that dynamically allocates bandwidth in closed loop with user state and task demands [1].

### 6.4 Why Simulate Bionic Vision in Sighted Participants?

Because no large user base of implant recipients exists, clinical studies remain small and heterogeneous. SPV is therefore a widely used, cost-effective testbed for early-stage evaluation [7, 30, 35]. While sighted participants cannot model long-term neural adaptation, they enable controlled, within-subject comparisons [3]. Our use of psychophysically validated phosphene models moves beyond simplistic blob renderings [9, 12, 32, 47] and supports rapid prototyping before clinical deployment [4].

### 6.5 Limitations & Future Directions

Our study addressed a single navigation task in a controlled VR setting. Generalization to other tasks, environments, and users remains to be tested. Although our simulation incorporated fading and distortion, it cannot capture the full variability of real prosthesis users (e.g., electrode–retina interactions, cortical plasticity [29, 34]). A design limitation is that *Control* was not counter-balanced. We chose this to avoid carryover from semantic overlays; learning/order checks suggest minimal impact, but future work should counterbalance fully or interleave conditions. Finally, dynamic obstacles remain a challenge: none of the strategies significantly reduced collisions with moving objects, motivating motion-aware or adaptive encoding techniques.

Despite these limitations, our results show that semantic simplification—and especially temporal structuring—can reduce perceptual burden in prosthetic vision. Progress toward intelligent, adaptive systems [4, 31, 42] will require closing the loop between simulation and clinical deployment, ideally through collaborative studies with implanted users [13, 37]. Combining computer vision with adaptive, multimodal feedback could empower users to navigate complex environments with greater independence.

## 7 Conclusion

We compared two semantic preprocessing strategies for prosthetic vision: one highlighting all task-relevant objects simultaneously (*SemanticEdges*), the other sequencing them over time (*SemanticRaster*). Both improved wayfinding over a traditional baseline, with *SemanticEdges* aiding global awareness and *SemanticRaster* reducing collisions. These findings support temporally adaptive, task-informed encoding as a design principle for clutter-aware XR interfaces and next-generation prosthetic vision.

## Supplementary Material

Supplementary Material

## CCS Concepts

• Human-centered computing → Accessibility technologies; Virtual reality; • Computing methodologies → Image processing; Image representations; Scene understanding.
