# Supplementary material for: Static or Temporal? Semantic Scene Simplification to Aid Wayfinding in Immersive Simulations of Bionic Vision
Source: Proc ACM Symp Virtual Real Softw Technol. Author manuscript; Available in PMC 2026 Sep 22. (PMC13592922; doi:10.1145/3756884.3766003)
Supplement: Supplementary file 1 [file NIHMS2208750-supplement-Supplementary_Material.pdf]

A Collision Feedback System

To ensure reliable event logging, collisions were registered only once per obstacle encounter. When a collision occurred, a UI overlay instructed participants to step back at least 0.25 m in the indicated direction before continuing. The overlay also identified the collided object (e.g., "Fountain" or "Person, walking"), and spatialized sound provided additional feedback (Figure A1). This prevented repeated collision counts from minor lingering contacts and maintained consistent trial flow.

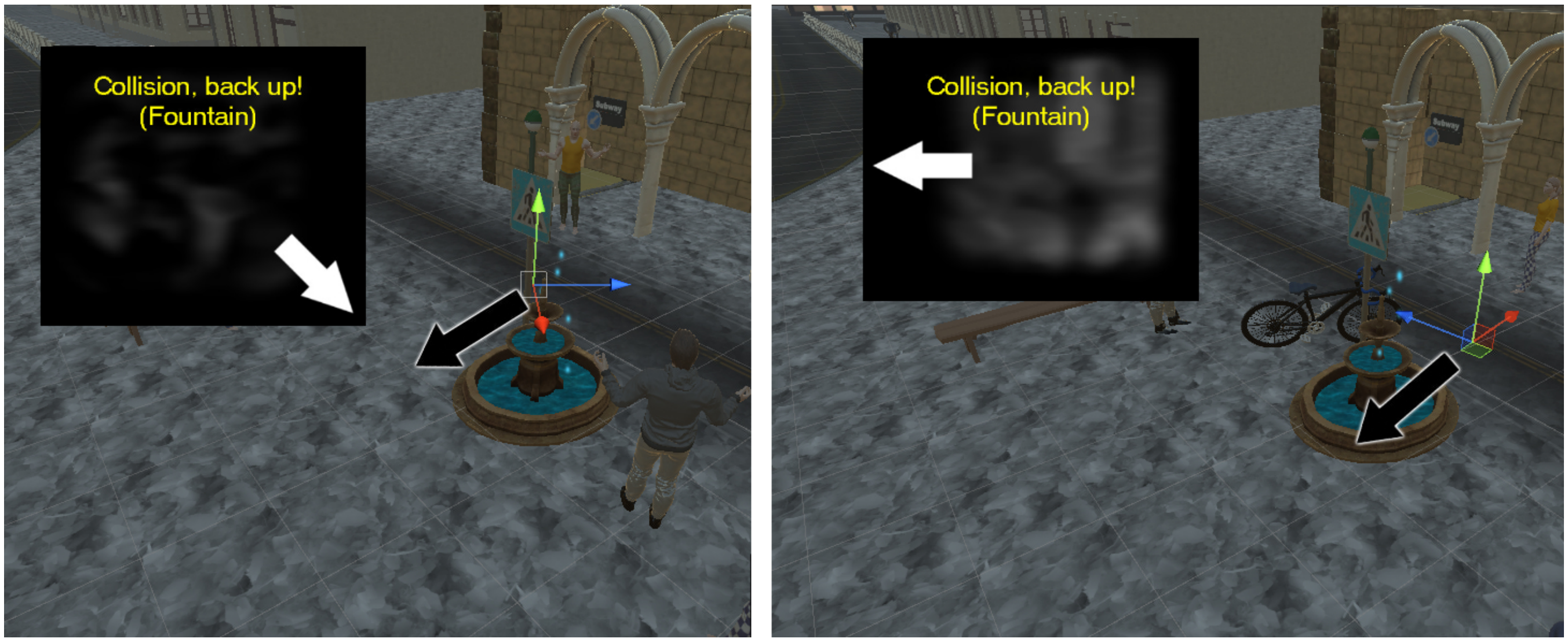

Figure A1: Collision feedback system. Participants had to move 0.25 m **to reset after a collision; an on-screen message and arrow indicated the direction, and the collided object was labeled.**

B Sensitivity/Power Analysis

We conducted Monte-Carlo power simulations matching the within-subject design (18 participants × 10 trials/condition × 3 conditions; $\alpha = .05$, two-sided).

For Success, we simulated a a logit model with subject random intercepts (variance= .229; ICC≈ .065), baseline $p_0 = .421$, and condition effects equal to the fitted odds ratios (*SmartEdges* OR= 1.87; *SmartRaster* OR= 1.33). Inference used GEE with exchangeable working correlation. Estimated power to detect a condition effect vs. Control was 0.83 (*SmartEdges*) and 0.29 (*SmartRaster*).

For moving collisions, we used a Poisson generator with baseline $\lambda_0 = .244$ and rate ratios (*SmartEdges* RR= 0.64; *SmartRaster* RR= 0.72). Subject random-effects variance was ≈ 0 (singular in the fitted model). Inference used GLM with cluster-robust (by-subject) SEs. Power was 0.44 (*SmartEdges*) and 0.30 (*SmartRaster*).

For total collisions, we used a Negative Binomial (NB2) generator with baseline $\lambda_0 = 2.62$, dispersion $\theta = 1.31$ (Var $= \mu + \mu^2/\theta$), and subject random-intercept variance 0.062 on the log scale; condition effects matched the fitted rate ratios (*SmartEdges* RR= 0.82; *SmartRaster* RR= 0.79). Inference again used GLM with cluster-robust SEs. Power was 0.44 (*SmartEdges*) and 0.57 (*SmartRaster*).

These results indicate adequate power for the primary success effect of SmartEdges and modest–moderate power for collision outcomes, consistent with observed p-values. Simulation code and seeds are provided in the repository.

C Eye Tracking Accuracy of the HTC Vive Pro

To assess the precision of the HTC Vive Pro's built-in eye tracker, $n = 30$ participants tracked a moving on-screen dot (∼ 2.4° visual angle) using their eyes. The dot moved randomly between four corners positioned halfway between the center and edges of the screen. It traversed the distance between points over 2.5 ± 0.5 seconds and remained stationary at each location for 1.5 seconds.

The angular error, defined as the distance between the dot's center and the user's gaze location, was measured every 0.1 s. Measurements were taken during both fixation (when the dot was stationary) and pursuit (when it was moving). Mean angular error was $1.904(2048)^\circ$ during fixation and $1.838(1660)^\circ$ during pursuit, with no significant difference between the two conditions (t-test for non-equal variances, $p > 0.27$).

Overall, 94.1% of measurements had an angular error below 5°, and 80% were below 3°. These results indicate that the HTC Vive Pro provides adequate precision for gaze-contingent rendering in SPV experiments (Figure A2).

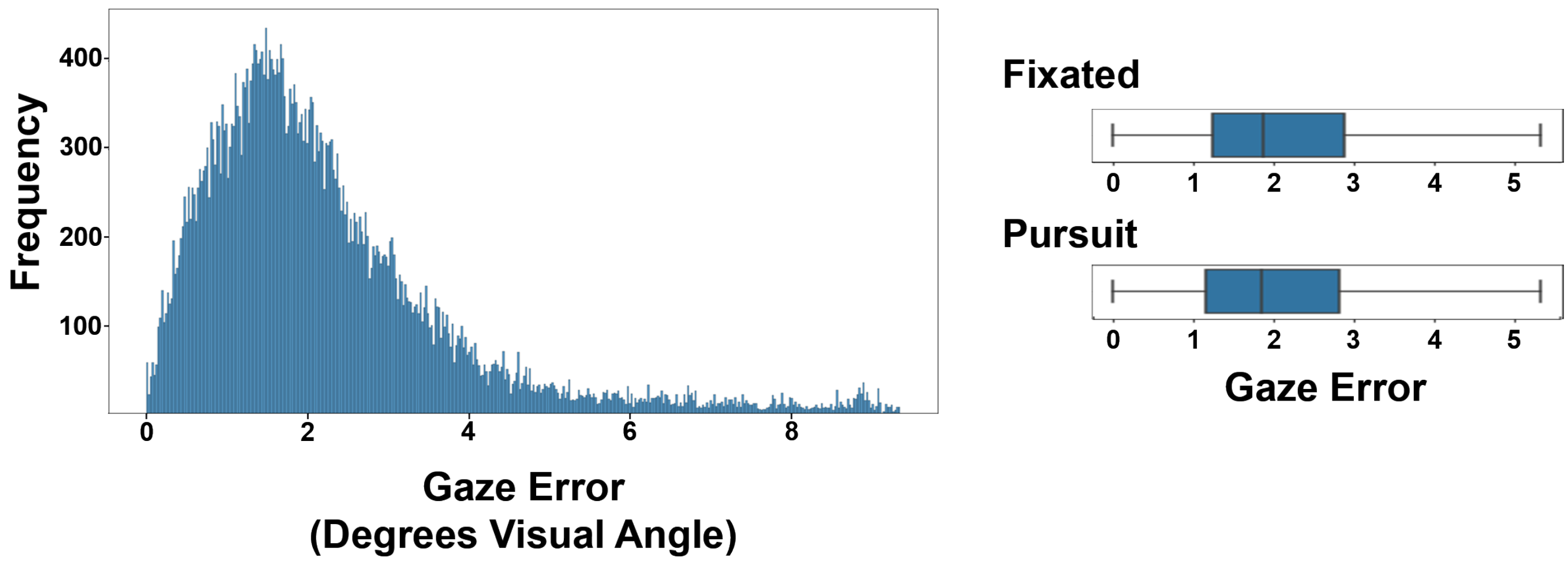

Figure A2: Eye tracking accuracy of the HTC Vive Pro. The histogram (left) shows the distribution of angular gaze error (degrees visual angle) across all measurements, with most errors falling below 5°. The boxplots (right) compare gaze error during fixation (when the dot was stationary) and pursuit (when the dot was moving). Mean errors were similar across conditions ($1.904(2048)^\circ$ for fixation and $1.838(1660)^\circ$ for pursuit), with no significant difference between the two (t-test, $p > 0.27$). Over 94% of measurements had an error below 5°, confirming the system's precision for gaze-contingent rendering.
